# Effects of Titanium Dioxide (TiO_2_) on Physico-Chemical Properties of Low-Density Polyethylene

**DOI:** 10.3390/polym16192788

**Published:** 2024-10-01

**Authors:** Peter P. Ndibewu, Tina E. Lefakane, Taki E. Netshiozwi

**Affiliations:** 1 Department of Chemistry, Tshwane University of Technology (TUT), Arcadia Campus, Arcadia, P.O. Box 56208, Pretoria 0001, South Africa; 2 Protechnik Laboratories, P.O. Box 8854, Pretoria 0001, South Africa

**Keywords:** Photocatalytic TiO_2_, low-density polyethylene, permeation, 2-chloroethyl ethyl sulphide

## Abstract

Hazardous chemicals are transported on rail and road networks. In the case of accidental spillage or terror attack, civilian and military first responders must approach the scene equipped with appropriate personal protective equipment. The plausible manufacturing of chemical protective polymer material, from photocatalyst anatase titanium dioxide (TiO_2_) doped low-density polyethylene (LDPE), for cost-effective durable lightweight protective garments against toxic chemicals such as 2-chloroethyl ethyl sulphide (CEES) was investigated. The photocatalytic effects on the physico-chemical properties, before and after ultraviolet (UV) light exposure were evaluated. TiO_2_ (0, 5, 10, 15% wt) doped LDPE films were extruded and characterized by SEM-EDX, TEM, tensile tester, DSC-TGA and permeation studies before and after exposure to UV light, respectively. Results revealed that tensile strength and thermal analysis showed an increasing shift, whilst CEES permeation times responded oppositely with a significant decrease from 127 min to 84 min due to the degradation of the polymer matrix for neat LDPE, before and after UV exposure. The TiO_2_-doped films showed an increasing shift in results obtained for physical properties as the doping concentration increased, before and after UV exposure. Relating to chemical properties, the trend was the inverse of the physical properties. The 15% TiO_2_-doped film showed improved permeation times only when the photocatalytic TiO_2_ was activated. However, 5% TiO_2_-doped film exceptionally maintained better permeation times before and after UV exposure demonstrating better resistance against CEES permeation.

## 1. Introduction

In this study, the interest focused on investigating the effects of titanium dioxide (anatase) (TiO_2_) on the physical and permeation properties of low-density polyethylene (LDPE) films. LDPE has low tensile and compressive strength [1]. LDPE films have beneficial physical properties that include good moisture barrier, low melting point, heat-sealable, chemically inertness and relatively high gas permeability [2]. LDPE films are inexpensive than most thermoplastic polymer films and, hence, are widely used in various applications such as cling film, sandwich bags, squeezable bottles, plastic grocery bags and so forth [3,4]. The presence of a high number of branched chains in LDPE is responsible for its low density. Although LDPE is chemically inert at room temperature, it can be attacked by strong oxidizing agents, and most solvents are known to cause its softening or swelling [5]. However, its uses are limited due to several drawbacks, such as, low strength, stiffness, and poor heat resistance. At 95 °C, it is reported that LDPE remains intact for a short period, whereas at 80 °C it is durable for longer hours. The advantage of LDPE arises from its waxy and low melting point properties, which makes LDPE preferred for further development, over other polymer types. They offer good opportunities for tailor-made polymer films with new properties to meet the targeted needs [6]. Furthermore, developed films have been produced without causing a significant chemical modification on the polyethylene matrix [7]. However, depending on the additives used, these modifications may affect the dimensional stability, crystallinity, mechanical and other physico-chemical properties of polymers [8].

TiO_2_ was selected as an additive to modify LDPE because it is reportedly stable under harsh processing conditions [9]. It is chemically inert, resistant to corrosion, inexpensive and is well-known as a photocatalyst [10]. When TiO_2_ photocatalyst is irradiated with an energy higher than its band gap energy (3.2 eV), electrons are promoted from the valence band to the conduction band, leaving positive electron holes in the valence bands [11,12]. These photo-generated electrons and positive electron holes are unstable and very reactive. An electron can travel to the surface of TiO_2_ and react with absorbed oxygen (O_2_) to form a very reactive superoxide radical ion (O_2_)^●−^ which quickly reacts with water to form a hydroperoxy radical (HO_2_)^●^. According to Amin et al. (1975), hydroperoxy radicals are key intermediates in photodegradation because they decompose to produce radicals that can abstract hydrogen atoms from the polymer, and thus initiate photooxidation [12,13]. According to Tofa et al. (2019), the generated hydroxyl and superoxide radicals from the TiO_2_ initiate degradation at weak spots of the long polymer chain [14]. The effects of photodegradation are manifested in changes in the physical, mechanical and chemical properties of the exposed polymers, these changes are dependent on the intensity and the duration of the exposure [15,16,17].

Synthetic polymers like polyethylene ((C_2_H_4_)n), have provided durability at low cost. The flexibility of polyethylene to be developed into different types of products was subject to its various densities. Examples of the polyethylene polymer densities are classified as follows: linear low-density polyethylene (LLDPE), LDPE, high-density polyethylene (HDPE) and ultra-high molecular weight polyethylene (UHMWPE) [1]. Thus, taking advantage of LDPE flexibility enables products such as chemical-resistant protective clothing (suits and aprons) to be developed. Khalil (2015), Ndibewu et al. (2016) and others have reported that chemical protective clothing (CPC) is manufactured from materials consisting of multiple layers of the same or different polymer films [18,19]. CPC are classified according to their material composition as air-permeable, semi-permeable, impermeable as well as selective protective materials [20].

When focusing on the protective materials consisting of polymeric layers, most are found within the impermeable and selective protective materials. These materials are utilized in the production of CPC which are ranked according to their levels of protection against chemical hazards. Their protection levels are ranked from levels A to D corresponding to chemical toxicity exposure from high to low, respectively. Factors determining a chemical hazard include assessing the level of risk, quantity of chemicals, exposure time and the level of chemical toxicity [18,19]. Hazardous chemicals are available in most industries and are being transported on public road networks daily. The purpose of the CPC is to protect the wearer from being in direct contact with hazardous chemicals, for example, accidental spillages during bulk transportation of pesticides and herbicides used in crop farming and chlorine used in wastewater treatment plants by tankers [19].

The objective of this study was to attempt to develop an alternative cheaper polymer material that will resist permeation of highly toxic chemicals such as bis(2-chloroethyl)sulphide (HD) to the same degree as the more costly polymer materials used to develop the commercial chemical and biological protective suits. Dolez et al. (2022) and other researchers, summarized different materials used in the manufacturing of impermeable chemical protective clothing (CPC) such as butyl rubber, neoprene, plastic films like polyethylene (PE), chlorinated PE, polytetrafluoroethylene (PTFE), polyvinyl chloride (PVC) and polyvinylidene chloride (PVDC) [20,21,22]. Some of the renowned manufacturers of the impermeable CPC are, for example, DuPont, Draeger, Kappler and others), utilizing multi-layered barrier films consisting of HDPE, D-mex (five-layers), and polyolefins, respectively [23,24,25]. It has been reported that protective materials can’t protect against all types of chemicals, meaning materials will be selective to specific chemicals and of course their intended function. Therefore, upon selection of the CPC, applicable permeation times (or rates) for chemical warfare agents (HD) differ, ranging from as little as 10 min to most common time frames of 8 h or even into longer times of at least 480 h [23]. Bis(2-chloroethyl)sulphide, a very toxic chemical warfare agent is prohibited by the Chemical Weapons Convention, and it is, therefore, not commercially available. However, there is HD simulant, namely 2-chloroethyl ethyl sulfide (CEES), which is commercially available. It was selected in this study to evaluate the permeation of TiO_2_-doped LDPE polymers produced in this study. Therefore, it is anticipated that if the objective of this study is achieved, the produced TiO_2_-doped LDPE polymer will be utilized to manufacture cost-effective durable lightweight protective garments which will offer protection against possible exposure to toxic chemicals, including HD [19,26,27].

According to Khalil and co-workers [18,28], there are four possible interactions between a chemical agent and a chemical protective material, which are:

(a) Chemical degradation–partial or selective breakdown of the polymer due to the presence of a chemical or various chemical rendering the non-toxic or less toxic;
(b) Chemical penetration–chemical diffuses through the wicking or imperfections and closures in impermeable structures;
(c) Chemical permeation–this is a molecular path of chemicals through the material of the structure; and
(d) Chemical evaporation–the exposed chemical may depend on their repellency, evaporation or the vapour of the chemical droplet may dissipate into the atmosphere or permeate through the CPC.

The chemical degradation and penetration are anticipated to affect the TiO_2_-doped films under study, especially after being exposed to UV light. The films may either degrade due to the interaction of UV light by breaking down the chemical composition or create cracks which may lead to possible penetration crevices for the chemical. Also, a highly eminent possibility is the prediction of an exposed chemical following the diffusion principle through the produced films. Regarding chemical evaporation, the chosen cup test method is performed under monitored environmental conditions and a test chamber is enclosed to prevent the evaporation of the exposed chemical.

In this study, it was postulated that when TiO_2_-doped LDPE films are exposed to ultraviolet exposure, their surfaces may be highly reactive which would serve as a physical barrier and may facilitate chemical reactions between the surface and molecules of a permeating chemical agent, CEES. The observed effects of TiO_2_-doped LDPE films (0, 5, 10 and 15% wt), on crystallinity, physical properties and permeation times before and after UV exposure will be discussed in the next sections. The objective is to investigate the plausible manufacturing of chemical protective polymer material, from photocatalyst anatase TiO_2_-doped LDPE films, for cost-effective durable lightweight protective garments against toxic chemicals such as 2-chloroethyl ethyl sulphide (CEES).

## 2. Materials and Methods

### 2.1. Materials

The following chemicals, materials, equipment and instruments, including their software, were utilized in the experimental work:

#### 2.1.1. Chemicals

- Titanium dioxide (TiO_2_)—>99% purity; Anatase; Merck, Johannesburg, South Africa;
- 2-Chloroethyl ethyl sulphide (CEES)—>99% purity, Merck, Johannesburg, South Africa;
- Nitrogen gas—99.999% purity, Afrox supplier, Germiston, South Africa.

#### 2.1.2. Materials

- LDPE pellets—SASOL, Modderfontein, South Africa.

#### 2.1.3. Equipment and Instruments

- Co-rotating twin screw extruder—LabTech Engineering, Phraeksa, Thailand;
- Film blowing twin screw extruder—Nanjing Extrusion Machinery, Nanjing, China;
- Cryo-microtome—Leica UC7, Leica Microsystems, Wezlar, Germany;
- Scanning electron microscope coupled to an energy-dispersive X-ray spectrometer (SEM-EDS)—Zeiss FE-SEM 55VP, Zeiss, Jena, Germany;
- Transmission electron microscope (TEM)—JEM 1010, Joel, Tokyo, Japan;
- Simultaneous Thermal Analyzer (STA) 6000—Perkin Elmer, Waltham, MA, USA;
- Tensile tester—Instron 3345, Norwood, MA, USA;
- UV source (UV-B Lamp; 295–320 nm)—Philips, Amsterdam, The Netherlands.

#### 2.1.4. Software

- Pyris Software for STA 6000—Ver.13;
- Bluehill LE for Instron 3345—Ver.3.77.4940.

### 2.2. Methods

#### 2.2.1. Extrusion of LDPE Films

The extrusion was conducted using the co-rotating twin screw (LabTech Engineering, Thailand) with a diameter of 30 mm and length-to-diameter (L/D) ratio of 40. The raw materials, LDPE pellets with density of 0.921 g.cm^−3^ (purchased from SASOL, South Africa) with and without metal oxide (TiO_2_), were compounded at temperatures starting from 120 °C to 175 °C as per the optimization conditions over ten (10) different temperature zones. The oven-dried extrudates were film blown to the thickness of 150 µm using the 36 mm-twin-screw extruders (Nanjing Extrusion Machinery, China). Four films (30 cm wide and 3 m long) were prepared by incorporating TiO_2_ (>99%; anatase; Merck Chemicals, Germany) at different concentrations with LDPE pellets resulting in 0, 5, 10 and 15%wt doped LDPE films, respectively. The films are designated as follows: neat LDPE, 5% TiO_2_-doped film, 5% TiO_2_-doped film and 15% TiO_2_-doped film according to their respective doping %wt of TiO_2_.

#### 2.2.2. Morphology

About 1 cm^2^ of polymer films were sectioned with a cryo-microtome (Leica UC7) at the liquid nitrogen temperature (−196 °C). The cut samples were polished and coated with carbon because they possess the properties of a semiconductor. The dispersion of the TiO_2_ particles was evaluated using the scanning electron microscope coupled to an energy-dispersive X-ray spectrometer (SEM-EDS) (Zeiss FE-SEM, 55VP). Additionally, transmission electron microscope (TEM) (Joel, JEM 1010) was utilized to determine the dispersion and size of the TiO_2_ particles on the film surface.

#### 2.2.3. Determination of Thermal Properties

Strips of polymer films were fragmented into small pieces to fit into the thermal analyzer crucible. Approximately 10 mg of the sample was weighed by mass difference. The degree of crystallinity and mass loss were determined by analysis on the Simultaneous Thermal Analyzer (STA) 6000 (Perkin Elmer, Waltham, MA, USA) consisting of the dual testing capability of differential scanning calorimetry (DSC) as well as thermogravimetric analysis (TGA). The analysis (single run) was initiated by holding the temperature at 40 °C for 4 min, followed by temperature programming at 10 °C/min up to 200 °C and held for 2 min under nitrogen (99.999% purity, Afrox supplier) atmosphere and thereafter it was cooled down to 40 °C at 10 °C/min Also, for TGA, analysis was initiated by holding the temperature at 40 °C for 1 min, followed by a gradual increase to 900 °C at 10 °C/min, and held at 900 °C for 2 min under nitrogen flow.

#### 2.2.4. Tensile Testing

Following the standard test method, D412-16 (by American Society for Testing and Materials (ASTM)), dumbbell-shaped samples were cut out and conditioned in an environment of 23 ± 2 °C and humidity between 45–55% for at least 3 h before analysis. The samples (5 replicates/sample) were analyzed using a tensile tester (Instron 3345, Norwood, MA, USA) with a pulling rate of 500 mm/min and a load of 2 kN.

#### 2.2.5. Permeation Testing

Samples with a diameter of 40 mm, free of any deformations, were cut out and sandwiched between two chambers of the permeation-dedicated test glassware. In the bottom chamber, 3 mL of methyl red indicator solution was placed. The indicator solution pH was adjusted to pH 6.6 to show or reflect a yellow colour. In the top chamber, droplets (1 µL × 3) of the 2-chloroethyl ethyl sulphide (CEES) were spiked. The sandwiched samples were air-tightly sealed. Samples were analyzed in triplicates with a fourth sample being a control sample. Exposure time was recorded from the instant the first CEES droplet was spiked until there was a complete colour change in the indicator solution from yellow to pink, and that was recorded as a breakthrough or permeation time. The control sample was not spiked with CEES, but it was monitored concurrently with the spiked samples for any colour change which would indicate any interference between the sample film and the indicator solution. More importantly, only the first replicate to show colour change was captured as a critical indicator and thus their respective times were recorded whilst other replicates were observed for their inconsistent permeation time response to the CEES.

#### 2.2.6. UV Exposure Testing

Additional samples were prepared similarly for the tensile as well as permeation testing. They were reserved for UV exposure analysis. These samples were placed in the UV chamber and their positions were rotated, after every 48-h interval, for equal and maximum exposure to emitted radiation from the UV source (UV-B Lamp; 295–320 nm; Philips). Each sample was irradiated continuously for ten days (240 h). These samples are referred to as UV-exposed samples.

#### 2.2.7. Statistical Analysis

Data captured from the replicate results was recorded as the mean ± standard deviation. The data was evaluated using the one-way analysis of variance (ANOVA) and *p*-values ≤ 0.05 were considered significant.

## 3. Results and Discussion

### 3.1. Morphology Studies

**Figure 1:**
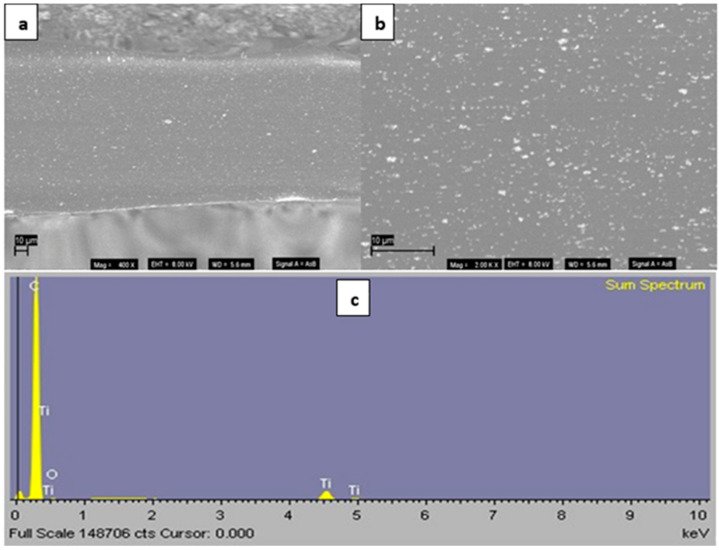
Distribution of TiO_2_ observed on the SEM images taken from the 10% TiO_2_-doped film before UV exposure: (**a**) Cross-section view, (**b**) Surface view, and (**c**) EDS spectrum.

**Figure 2:**
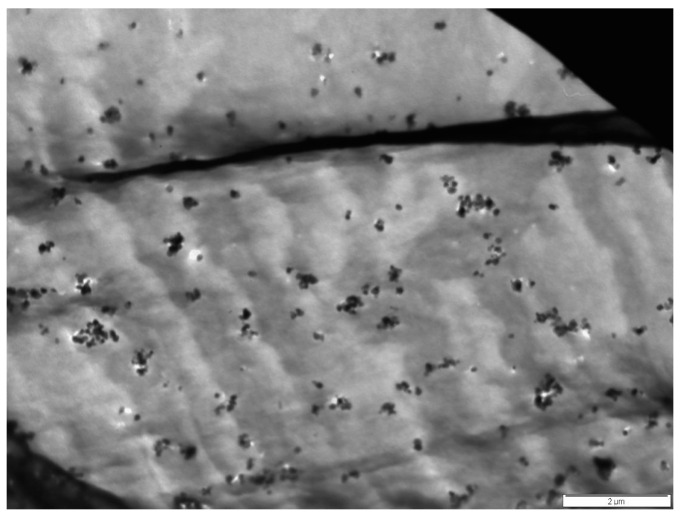
The TEM image capturing the distribution of the 10% TiO_2_ doped film.

The extrusion and film-blowing processes followed to produce polymer films, and their composites were partially automated. The weighing and feeding into the feeder involved manual operation, thus, inconsistency was highly likely. Therefore, the topography of the polymer films was assessed using SEM-EDS and TEM. The films were prepared under cryogenic conditions to inhibit possible deformation or cracking that may insinuate false topography. The distribution of the TiO_2_ particles is demonstrated on the surface as well as on the cross-section of the polymer. Distribution of agglomerated TiO_2_ particles observed on the SEM images taken from the 10% TiO_2_-doped film before UV exposure is shown in Figure 1. (a) Cross-section view, (b) Surface view, and (c) EDS spectrum. In addition, similar topography trends for 5% and 15% TiO_2_-doped film composites were achieved. This was complimented by the TEM micrograph confirming that the aggregated TiO_2_ particles represent a much smaller fraction of the 2 µm in dimensions, as shown in Figure 2. The micrograph shows fair to good distribution of agglomerated TiO_2_ particles in polymer matrix. In the cross-section micrograph (Figure 1a, the agglomerated TiO_2_ particles are distributed fairly even across the film thickness implying that the TiO_2_-doped LDPE films will present a ‘uniform’ sample. The other benefit of well-distributed agglomerated TiO_2_ particles is to promote even photocatalytic degradation across the area, especially after UV exposure [29].

The EDS spectrum in Figure 1c confirms the presence of the dominant elements, which are titanium (Ti), oxygen (O) and carbon (C). There were no traces of significant interferences observed. On observation, the produced films were as follows: Neat LDPE showed transparent smooth surface films; 5 and 15% TiO_2_-doped films showed white glossy smooth surfaces, whilst 10% TiO_2_-doped film showed a white non-glossy smooth surface. Therefore, neat LDPE and TiO_2_-doped polymer films were successfully produced, and further evaluations (physical and chemical studies) were conducted.

### 3.2. UV Exposure

Ultraviolet radiation consists of electromagnetic waves with a wavelength ranging from 100 to 400 nm. This is further divided into three regions namely, UV-C ranging from 100 to 280 nm, UV-B from 280 to 315 nm and UV-A from 315 to 400 nm wavelength [12,16,17]. UV-A generated from the sun, completely reaches the earth, while all the UV-C is completely absorbed by the ozone layer. The UV-B (280–315 nm) has an energy of 426–380 KJ.mol^−1^ which is destructive for organic compounds [17]. Nonetheless, most of the higher energetic part of UV-B (280–295 nm) is absorbed by the ozone layer, and only small amounts manage to reach the earth [12,16,17]. UV radiation causes photooxidative degradation of polymers, which affects the physical, mechanical and chemical properties of polymers [30]. This observation combined with the fact that TiO_2_ is a photocatalyst prompted the postulation that when TiO_2_-doped LDPE films are exposed to UV light, a reactive organic radical might be produced and that will react with the polymer leading to either reinforcing or weakening some of its physico-chemical properties on the polymer matrix. Therefore, the effects of UV exposure on the films were determined by observing the results shift of crystallinity, mass loss, tensile properties and permeation of 2-chloroethyl ethyl sulphide (CEES) through the polymer films.

### 3.3. Thermal Analysis

**Figure 3:**
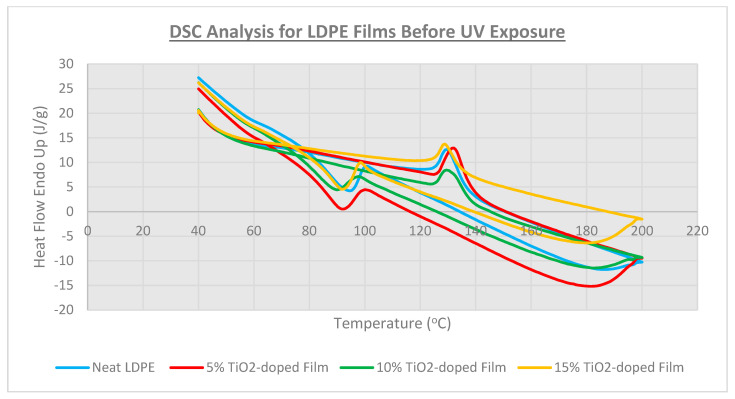
DSC thermograms of LDPE films before UV exposure.

**Figure 4:**
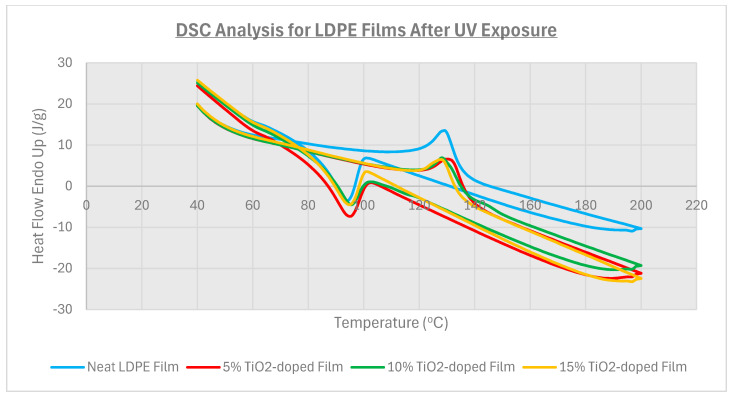
DSC thermograms of LDPE films after UV exposure.

**Figure 5:**
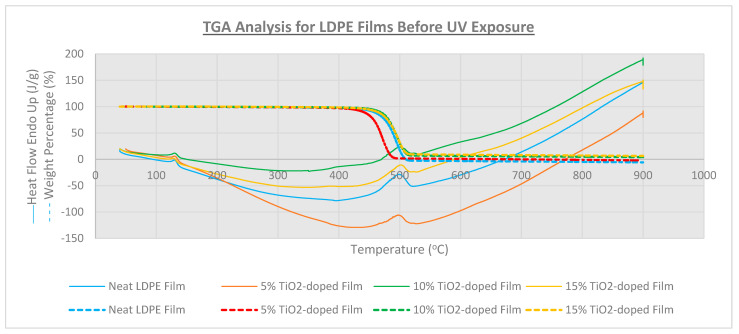
TGA thermograms of LDPE films before UV exposure.

**Figure 6:**
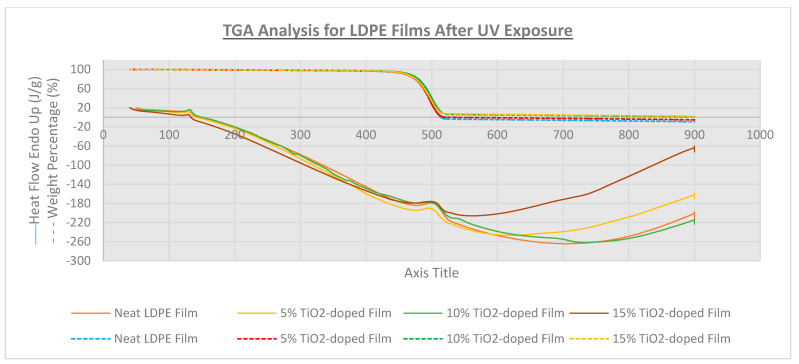
TGA thermograms of LDPE films after UV exposure.

According to Poh et al. (2022), the energy required to melt pure or 100% polyethylene is 293 J/g [31]. The degree of crystallinity is determined by calculating the ratio of measured enthalpy against that of pure polyethylene enthalpy. The obtained results are summarized in Table 1. It has been reported by other co-workers [32] that LDPE is semi-crystalline and its degree of crystallinity ranges between 30 and 50%. The reported degree of crystallinity for other closely related polymers like high-density polyethylene, polypropylene, and polyethylene terephthalate are 80–90%, 30–50% and 10–30%, respectively. The neat LDPE film produced in this study was below the lowest limit of semi-crystallinity at 13.7 and 17.7% before and after UV exposure, and thus, it is eligible to be defined as amorphous (molecular chains are randomly oriented). A summary of results obtained from the neat LDPE as well as the TiO_2_-doped films is shown in Table 1. The thermograms of produced LDPE films are shown in Figure 3 and Figure 4 for DSC results and followed by Figure 5 and Figure 6 for TGA results.

Factors like physical or thermal treatments tend to influence the crystallinity of the polymer. This was observed in this study whereby the addition of TiO_2_ to the neat LDPE surface, resulted in its degree of crystallinity decreasing with the increasing TiO_2_ concentration from 13.3% to 9.6% before UV exposure. After UV exposure, the degree of crystallinity for TiO_2_-doped films was reduced from 18.8% to 13.7%, with the 15% TiO_2_-doped film declining to the level of the untreated neat LDPE film (13.7%, before UV exposure). As reported by Tofa et al. (2019), the generated hydroxyl and superoxide radicals from the TiO_2_ initiate degradation at weak spots of the long polymer chain, [14] hence the effect of UV irradiation degrades the new structure and brings it to its original structure of LDPE.

The melt onset (Tc) and melt peak (Tm) temperature results obtained from the DSC curves were also reported by Nguyen et al. (2018). Li et al. (2019) [33,34]. However, Li et al. (2019) achieved a degree of crystallinity for neat LDPE at approximately 39% whereas Nguyen achieved below the semi-crystalline level at approximately 24% before treatment and an increase to 27% after treatment. Therefore, it is not a unique scenario to have achieved results fitting in the amorphous region of the LDPE.

The Tc and Tm increased with the increasing concentration of the TiO_2_ before and after UV exposure. Interestingly, with neat LDPE, the Tc and Tm did not show any significant change before and after UV exposure. The only observed change was its degree of crystallinity changing from 13.7% to 17.7% due to the weakening created by the damage from the UV light.

Ironically, 5% TiO_2_-doped film did not show a change in Tm and yet its Tc and degree of crystallinity showed a change in results. The presence of 5% TiO_2_ seemed to be miniscule to play a significant role in LDPE film. Nguyen et al. (2018) reported that the melting temperatures of the films slightly increase by about 1–3 °C after degradation, if compared to their respective unexposed films [33]. A similar trend was observed in this study. It was further reported that this could be because of the increasing degree of crystallinity after UV exposure whereby TiO_2_ may exist as a nucleating agent for the crystallization of LDPE [33].

The results obtained from TGA are included in Table 1 and displayed in Figure 5 and Figure 6. The presence of 5% TiO_2_ in the LDPE film does not show any significant effect on the film in terms of the thermogravimetric analysis results as the neat LDPE and 5% TiO_2_-doped film show complete mass loss at the end of the analysis and the same degradation temperature at 478 °C and 475 °C, before and after UV exposure, respectively. As the doping concentration increases, the incomplete mass loss is observed representing the presence of metal oxide, TiO_2_, content. The mass loss was moderate (95.9% and 93.6% for 10 and 15% TiO_2_-doped films) before UV exposure when compared to almost complete mass loss after UV exposure (99 and 98.2% for 10 and 15% TiO_2_-doped films). During UV exposure, the films undergo physico-chemical transformation which portrays different properties and thus result in lower residues of metal oxide, O-Ti-O, content. Interestingly the photocatalytic effect is observed with the degradation temperatures decreasing by mere 0.6% except for 10% TiO_2_-doped film, showing a decrease of 1.6% (482 °C to 474 °C) for results obtained before and after UV exposure. Also, it is anticipated that carbon chain of the LDPE films could be decomposed into shorter chains with reduced molecular weight, leading to a decrease of thermal stability [33,35], thus the 0.6% degradation temperature shift.

### 3.4. Tensile Properties

The physical properties’ results of polymer films before UV exposure are presented in Table 2.

From the data presented in Table 2, it was observed that there were slight variations in extruded film thickness; therefore, this was compensated by normalization of thickness to 150 µm for better comparison of results. The tensile strength of the neat LDPE at 8.29 ± 1.8 MPa was higher than the 5% TiO_2_-doped films at 7.86 ± 1.2 MPa which was attributed to the slight stability change of the presence of the 5% TiO_2_ amount. Sadrnia et al. (2021) have reported similar results that showed about 29% decline in neat polyvinyl alcohol (PVA) film with a tensile strength of 25.69 ± 6.08 MPa, whereas 1%wt TiO_2_/PVA film had its tensile strength reduced by 26% to 19.03 ± 3.01 MPa [36].

**Figure 7:**
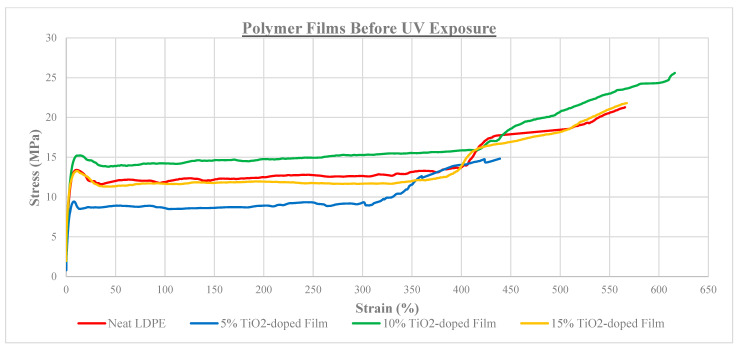
Stress-strain curve of the polymer films before UV exposure.

The introduction of 10 and 15%wt TiO_2_ onto LDPE films resulted in an improvement of tensile strength to 10.6 ± 1.0 MPa and 11.5 ± 1.0 MPa when compared to the neat LDPE (8.29 ± 1.6 MPa). This was an indication that the reinforcement of TiO_2_ on LDPE films had a significant (*p* < 0.05) effect on the tensile strength of the produced films from 10% TiO_2_ doping concentration. This observation is clearly illustrated in the graphical results of the stress-strain curves shown in Figure 7. Similarly, strain and elastic modulus properties of the extruded polymer films followed the same trend as that shown by the tensile strength properties Table 2, respectively.

**Figure 8:**
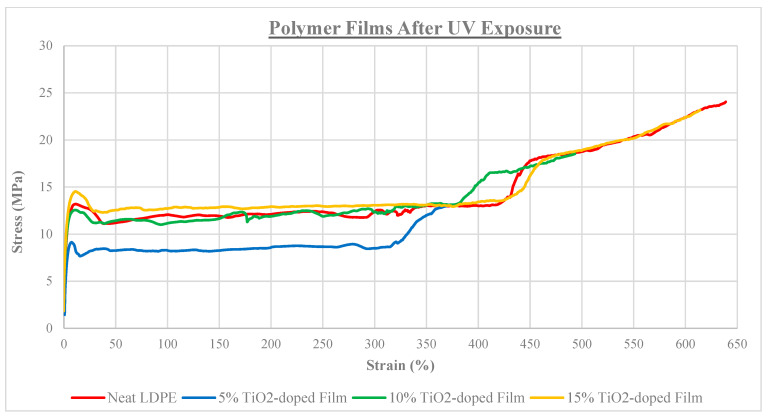
Stress-strain curve of the polymer films after UV exposure.

The UV-exposed neat LDPE film showed slightly higher tensile strength results of 9.67 ± 1.7 MPa (Table 3) when compared to the unexposed neat LDPE, with 8.29 ± 1.8 MPa (Table 2). Results of physical properties of extruded polymer films after UV exposure are shown in Table 3 and their stress-strain curves are shown in Figure 8.

The assumption was that the exposure to UV light should have a maximum impact on the neat LDPE, because of the polymer degradation induced by UV exposure. The results in Table 3 showed that the tensile strength of TiO_2_ doped films increased with increasing concentration of TiO_2_, even though the increase starting mark (7.34 ± 0.7 MPa) was far below the level of neat LDPE film (9.67 ± 1.7 MPa). Since TiO_2_ is a photocatalyst, the exposure of these films to UV light was expected to initiate degradation, which may result in weakening the polymer structural framework [37]. Furthermore, it was hypothesized that the tensile strength of the TiO_2_ doped films would show a decrease with increasing concentration of TiO_2_, due to the presence of TiO_2_ weakening the structural framework of the amorphous LDPE film however, the results showed the opposite, Figure 8.

**Figure 9:**
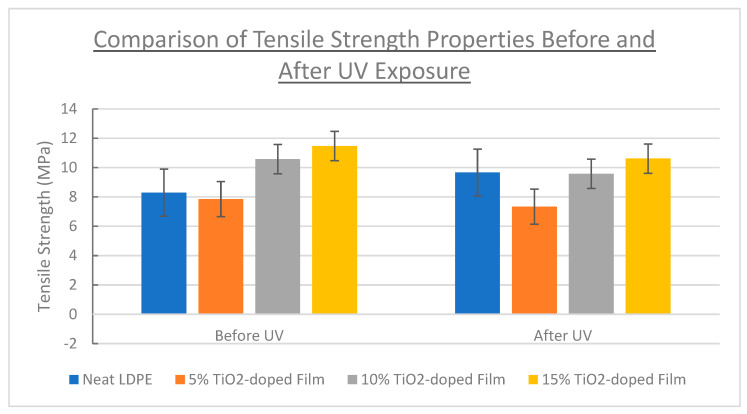
Comparison chart of tensile properties of the polymer films.

The tensile strength results before and after UV exposure were compared and are graphically represented in Figure 9. These comparative results showed that there were slight decreases in the tensile strength before and after UV exposure of all the films. The reinforcement of TiO_2_ doping showed a significant change from the 10% TiO_2_-doped film before UV exposure whereas a significant change was observed with the 15% TiO_2_-doped film, after the UV exposure. Activation of TiO_2_ by photolysis did not attribute to much improved properties as anticipated. It was reported that the decrease in tensile properties could be due to the increase in the brittleness which subsequently increases the cross-linking of the films after UV exposure [33].

When comparing the tensile strength of each film before and after UV exposure, the 5% TiO_2_-doped film showed a tensile strength of 7.86 ± 1.2 MPa which slightly decreased to 7.34 ± 0.7 MPa after the UV exposure. Thus, the reduction in tensile strength was expected as per the postulation that when a polymer is exposed to UV light there is a weakening in its structural framework. This result was similar to the observations by Zhao et al. (2015) that the rate of polymer framework weakening is dependent on the intensity of the UV light, as well as the duration of exposure [38].

### 3.5. Permeation Studies

Typically, LDPE films are composed of crystalline and permeable amorphous regions through which the permeate is believed to travel [39]. To further substantiate the above hypothesis, according to Mao (2008), polyethylene is modelled as a semi-crystalline polymer consisting of crystalline and amorphous zones [40]. The amorphous zones are made of polymer web-like structures where there are pinholes, through which the permeate molecules can diffuse. The crystalline zones act as impermeable barriers for sorption and diffusion, unless if the permeate at high concentrations causes it to swell, and thus it may result in an increased diffusion coefficient for the permeating component [26,41].

**Scheme 1:**
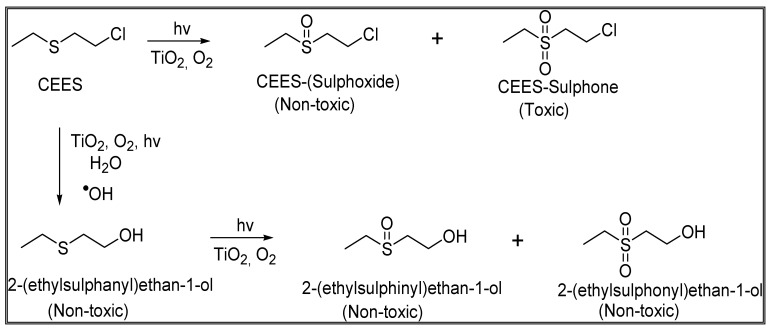
Reaction scheme illustrating possible products of CEES in the presence of TiO_2_, UV and Oxygen in the absence and the presence of moisture.

In this study, it was postulated that TiO_2_-doped LDPE film with its photocatalytic property will initiate a chemical reaction between the challenge-tested chemical, CEES, and the film surface during the permeation process. This prompted the postulation that TiO_2_ molecules embedded in the least crystalline regions of doped polymers will react with the permeating CEES molecules. Therefore, such reactions, in an environment where there is no moisture, would result in the oxidation of only the sulphur atom of the CEES to the corresponding sulphoxide and sulphone products, as illustrated in Scheme 1 [42,43]. The activation of TiO_2_ by exposure to UV light was anticipated to promote either photodegradation or complex oxidation reaction that may inhibit CEES permeation by oxidizing it to the corresponding non-toxic 2-chloroethyl ethyl sulphoxide by-products [44]. However, this activation might also result in the promotion of the polymer matrix degradation, thus, resulting in poor material towards resistance against a permeating chemical.

Alternatively, in the presence of moisture, the electrons produced from the photocatalyst, TiO_2_, may react with water molecule to produce hydroxyl radicals that could substitute chlorine atoms in CEES to produce the corresponding 2-(ethylsulphanyl)ethan-1-ol. This may be followed by sequential oxidation of sulphur atoms to form 2-(ethylsulphinyl)ethan-1-ol and 2-(ethylsulphonyl)ethan-1-ol, respectively [42]. The above reactions are normally carried in liquid media, and in this study, it was performed on a solid support in a closed chamber (without moisture) and the reaction rate will depend on the rate of permeation of CEES.

It was assumed that the formation of reaction products shown in Scheme 1, will disrupt the permeation process of CEES and result in the most concentrated, 15% TiO_2_-doped film offering more resistance to the permeate by reacting with more molecules of CEES. Surprisingly, this postulation was contradicted by the experimental results (Table 4) obtained before UV exposure.

**Figure 10:**
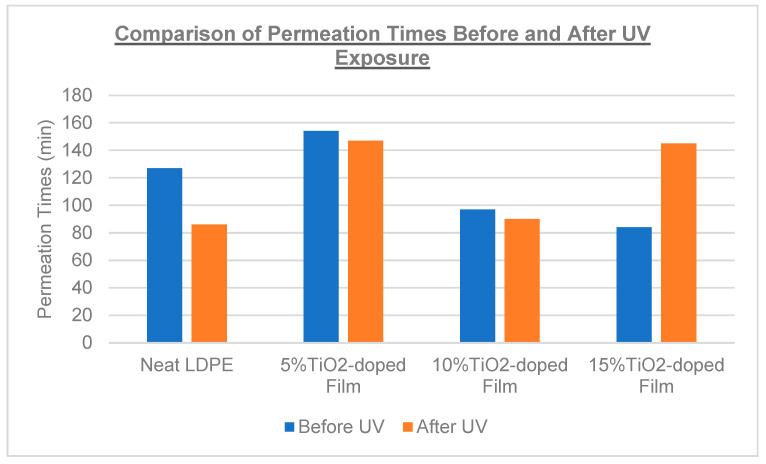
Comparison chart of the permeation times of the polymer films.

The 15% TiO_2_-doped film showed the shortest permeation time of 84 min (Table 4), whereas the neat LDPE film had a better permeation time of 127 min As the doped amount of TiO_2_ decreased, the permeation times were longer. However, the 5% TiO_2_-doped film performed better than the neat LDPE by at least 154 min Neat LDPE and 15% TiO_2_-doped film marked a significant difference in permeation times between the films before and after UV exposure as illustrated in Figure 10. There was no difference between the 5% and 10% TiO_2_-doped films before and after UV exposure. The effect of exposure to UV was more visible in neat LDPE and 15% TiO_2_-doped films. This negates the fact that titanium dioxide (TiO_2_), is solely responsible for the degradation.

Surprisingly, UV exposure had remarkably reduced the permeation time of neat LDPE, from 127 min of unexposed film to 86 min after exposure. In this instance, the UV exposure of the neat LDPE resulted in photodegradation of the film, which is shown by the CEES molecules permeating easily through the polymer matrix.

The TiO_2_ played a significant (*p* < 0.05) role in protecting the 5% and 10% TiO_2_-doped films from the UV light’s structural damage when compared to the neat LDPE, which had its permeation time reduced by 41 min (approximately 32%) after the UV exposure. Other researchers, Ergerton et al. (2011) and Yang et al. (2010), have reported that TiO_2_ may protect the polymer from photodegradation and lengthen its lifetime [45,46]. After UV exposure, the 15% TiO_2_-doped film had its permeation time prolonged from 84 min to 145 min However, the initial postulation was that the 15% TiO_2_-doped film would yield the lowest permeation time after UV exposure, because it was assumed that the UV exposure would contribute to damaging the film matrix structure, which might result in cracks and pinholes, thus, enabling easier permeation of CEES molecules. Therefore, the exposure of 15% TiO_2_-doped film to UV light affected the film by increasing the crystalline zones when compared to the 15% TiO_2_-doped film before UV exposure. The doped amount (15% TiO_2_) seemed to be activated and created a platform suitable to oxidize the CEES into non-toxic CEES-Sulphoxide, hence the permeation time was delayed to 145 min

## 4. Conclusions

The conventional extrusion process was applied successfully to produce the desired neat LDPE and TiO_2_-doped films. The SEM-EDS confirmed the presence of a fairly even distribution of the agglomerated TiO_2_ particles on the surface as well as across the film width. The EDS eliminated the presence of other contaminants during extrusion processes and thus confirmed the presence of only expected elements, such as titanium, carbon and oxygen. TEM micrograph of the 10% TiO_2_-doped film also demonstrated the fair distribution of agglomerated TiO_2_ particles, which were studied under high magnification that indicated that the agglomerated TiO_2_ particles were a minuscule fraction in size if compared with the 2 µm scale provided.

The effect of physical and thermal treatment during the processing of LDPE doped films was assessed by evaluating the crystallinity and mass loss shift. Surprisingly, the least TiO_2_-doped LDPE films showed a significant shift of crystallinity (13.3% to 18.8%) and the doping effect did have a significant impact (*p* < 0.05) on improving crystallinity as all doped films presented crystallinity above the neat LDPE results (>13.7%) before UV exposure. It was assumed that with the increasing doping concentrations, there would be increasing resistance of CEES to permeate the films. However, the obtained results showed that only the 5% TiO_2_-doped film offered better resistance towards the permeation of CEES when compared to the neat LDPE film before and after UV exposure. The LDPE films doped with 10% and 15% TiO_2_ offered the least resistance towards the CEES permeation, instead, the neat LDPE offered better resistance than these two (10% and 15%) TiO_2_-doped films before UV exposure.

For the 5% TiO_2_-doped film, a decrease in tensile strength, strain and elastic modulus from the benchmark of neat LDPE of 8.29 ± 1.8 MPa, 370 ± 88% and 5.5 ± 0.4 MPa, respectively, was observed. On increasing the TiO_2_ doping concentration from 5% to 15%, there was a gradual increase in physical properties performance to 11.5 ± 1.0 MPa, 405 ± 48% and 8.7 ± 0.6 MPa for tensile strength, strain and elastic modulus, respectively. A similar pattern of results is observed even after UV exposure.

After UV exposure, an eminent effect was observed on the crystallinity with results at approximately 17.7%, which represented amorphous films were within the same range of neat LDPE film. The UV exposure activated TiO_2,_ and it was manifested by the increased permeation times of the 15% doped film observed before (84 min) and after (145 min) UV exposure. Thus, the evidence of photodegradation was observed on neat LDPE by the increase in crystallinity (13.7% to 17.7%) and tensile strength (8.29 ± 1.8 MPa to 9.67 ± 1.7 MPa), with a decrease in permeation times (127 min to 86 min) when compared to before and after the UV exposure. This was based on the disorientation of the carbon chain in the polymer matrix resulting in shorter chains with reduced molecular weight that led to a decrease of their thermal stability. However, the 5% and 10% TiO_2_-doped films did not show similar UV stimulation, they were within the same range as before UV exposure. The 15% TiO_2_-doped also showed an interesting trend, after UV stimulation, its crystallinity resulted in 13.7% like the unexposed neat LDPE, and its permeation time inclined sharply to 145 min

Therefore, the doping effects of TiO_2_ on the neat LDPE were more effective on the 15% TiO_2_-doped film where the physical properties were significantly improved before and after UV exposure. With respect to the improvement of the chemical permeation improvement, 15% TiO_2_-doped film showed improved permeation times only when the photocatalytic TiO_2_ was activated. However, 5% TiO_2_-doped film exceptionally maintained better permeation times before and after UV exposure demonstrating better resistance against CEES permeation.

## Figures and Tables

**Table 1:** Results of Thermal Analysis.

Film Identity	Melt Onset T_c_ (°C)	Melt Peak Temp. T_m_ (°C)	Enthalpy (J/g)	Crystallinity (%)	Mass Loss (%)	Degradation Temp. (°C)
**Before UV Exposure**						
Neat LDPE	95.9	109.7	40	13.7	-	478
5% TiO_2_-doped	93.7	112.1	39	13.3	-	478
10% TiO_2_-doped	91.8	111.0	30	10.2	95.9	482
15% TiO_2_-doped	93.7	109.1	28	9.6	93.6	482
**After UV Exposure**						
Neat LDPE	95.9	109.9	52	17.7	-	475
5% TiO_2_-doped	96.8	112.1	55	18.8	-	475
10% TiO_2_-doped	97.2	108.8	46	15.8	99.0	474
15% TiO_2_-doped	95.9	108.5	40	13.7	98.2	479

**Table 2:** Physical Properties’ Results of Extruded Polymer Films Before UV Exposure.

Film Identity	Thickness (µm)	* Tensile Strength (MPa)	* Strain (%)	* Elastic Modulus (MPa)
Neat LDPE	200	8.29 ± 1.8	370 ± 88	5.5 ± 0.4
5% TiO_2_-doped	200	7.86 ± 1.2	367 ± 51	5.4 ± 1.0
10% TiO_2_-doped	150	10.6 ± 1.0	449 ± 62	7.6 ± 0.7
15% TiO_2_-doped	140	11.5 ± 1.0	405 ± 48	8.7 ± 0.6

* Results are normalized to 150 µm thickness.

**Table 3:** Physical Properties’ Results of Extruded Polymer Films After UV Exposure.

Film Identity	Thickness µm	* Tensile Strength (MPa)	* Strain %	* Elastic Modulus (MPa)
Neat LDPE	170	9.67 ± 1.7	438 ± 57	6.3±0.9
5% TiO_2_-doped	200	7.34 ± 0.7	340 ± 36	5.5 ± 1.0
10% TiO_2_-doped	140	9.58 ± 1.2	424 ± 41	7.3 ± 0.7
15% TiO_2_-doped	130	10.6 ± 1.1	395 ± 30	8.5 ± 0.8

* Results are normalized to 150 µm thickness.

**Table 4:** Permeation Test Results Before and After UV Exposure.

Film Identity	Thickness (µm)	* Normalized Permeation Time (min)	Thickness (µm)	* Normalized Permeation Time (min)
	**Before UV Exposure**		**After UV Exposure**	
Neat LDPE	160	127	150	86
5% TiO_2_-doped	200	>154	210	>147
10% TiO_2_-doped	130	97	140	90
15% TiO_2_-doped	150	84	140	145

* Results are normalized to 150 µm thickness.

## Data Availability

The datasets presented in this article are not readily available because they are part of an ongoing research. Requests to access the datasets should be directed to the correspondence author, P.P.N.

## References

[1] Khanam P.N., AlMaadeed M.A.A. (2015). Processing and Characterization of Polyethylene-Based Composites. Adv. Manuf. Polym. Compos. Sci..

[2] Fenercioglu H., Polat S., Guglu M. (2018). Effects of Metal Nanoparticles on the Physical and Migration Properties of Low Density Polyethylene Films. J. Food. Eng..

[3] Dehbi A., Mourad A.I. (2016). Durability of Mono-Layer Versus Tri-Layers LDPE Films Used as Greenhouse Cover: Comparative Study. Arab. Chem..

[4] Gitchaiwat A., Likhitlert S., Kositchaiyong A., Israngkura K., Taptim K., Sombatsompop N. (2016). Uses of 2-hydroxypropyl–3-piperazinyl-quinoline carboxylic acid methacrylate and Terbutryn as algaecides in low-density polyethylene mulching films for agricultural applications. J. Plast. Film. Sheet..

[5] Ghatge S., Yang Y., Ahn J., Hur H.G. (2020). Biodegradation of polyethylene: A Brief Review. Appl. Biol. Chem..

[6] Sirocic A.P., Rescek A., Scetar M., Krehula L.K., Hrnjak-Murgic Z. (2014). Development of Low Density Polyethylene Nanocomposites Films For Packaging. Polym. Bull..

[7] Redhwi H.H., Siddiqui M.N., Andrady A.L., Hussain S. (2013). Durability of LDPE Nanocomposites with Clay, Silica, and Zinc Oxide–Part I: Mechanical Properties of the Nanocomposite Materials. J. Nanomater..

[8] Hung Y.C., Yemmireddy V.K. (2017). Using Photocatalyst Metal Oxide as Antimicrobial Surface Coatings to Ensure Food Safety-Opportunities and Challenges. Compr. Rev. Food. Sci. Food Safety.

[9] Esthappan S.K., Kuttappan S.K., Joseph R. (2012). Thermal and Mechanical Properties of Polypropylene/Titanium Dioxide Nanocomposite Fibers. Mater. Des..

[10] Yadav A., Vigneshwaran N., Prasad V., Kathe A.A., Raj S., Yadav D., Sundaramoorthy C. (2006). Functional Finishing in Cotton Fabrics Using Zinc Oxide Nanoparticle. Bull. Mater. Sci..

[11] Kemp T.J., McIntyre R.A. (2000). Mechanism of Action of Titanium Dioxide Pigment in the Photodegradation of Poly(vinyl Chloride) and Other Polymers. Prog. React. Kinet. Mec..

[12] Khalilova H.K., Hasanova S.A., Aliyev F.G. (2018). Photocatalytic Removal of Organic Pollutants from Industrial Wastewater Using TiO_2_ Catalyst. J. Environ. Prot..

[13] Amin M., Scott G., Tillekeratne L.M. (1975). Mechanism of the Photo-Initiation Process in the Polyethylene. Europ. Polym. J..

[14] Tofa T.S., Kunjali K.L., Paula S., Dytta J. (2019). Visible light photocatalytic Degradation of Microplastic Residues with Zinc Oxide Nanorods. Environ. Chem. Lett..

[15] Lipp-Symonowicz B., Sztajnowski S., Kardas I. (2006). Influence of UV Radiation on the Mechanical Properties of Polyamide and Polypropylene Fibres in Aspect of Their Restructuring. Autex. Res. J..

[16] Lee Q.Y., Li H. (2021). Photocatalytic Degradation of Plastic Waste: A Mini Review. Micromachines.

[17] Yousif E., Haddad R. (2013). Photodegradation and Photostabilization of Polymers, Especially Polystyrene: Review. SpringerPlus.

[18] Khalil E. (2015). A Technical Overview on Protective Clothing Against Chemical Hazards. AASCIT J. Chem..

[19] Ndibewu P.P., Ngobeni P., Lefakane T.E., Netshiozwi T.E., Parveen F. (2016). Recent Advances in Biopolymers.

[20] Dolez P.I., Marsha S., McQueen R.H. (2022). Fibers and Textiles for Personal Protective Equipment: Review of Recent Progress and Perspectives on Future Developments. Textiles.

[21] Bhuiyan M.A.R., Wang L., Shaid A., Shanks R.A., Ding J. (2019). Advances and applications of chemical protective clothing system. J. Ind. Text..

[22] Suhail M.S. (2017). An Overview of Chemical Protective Clothing. Int. J. Adv. Eng. Res. Sci..

[23] Dupont Personal Protection, Catalogue. https://www.dupont.co.in/content/dam/dupont/amer/us/en/personal-protection/public/documents/en/DuPont_Personal_Protection_Catalogue_APAC_EN.pdf.

[24] Draeger Specification Sheet CPS 7900. https://en.safetygas.com/chemical-cbrn-suit-cps7900.

[25] Kappler, Know What You Are Getting Into. https://shop.dqeready.com/content/documents/Kappler%20Level%20A%20HazMat%20Suit%20Instructions-User%20Information%20for%20Zytron%20Level%20A%20Garments.pdf.

[26] Kappert E.J., Raaijmakers M.J.T., Tempelman K., Cuperus F.P., Ogieglo W., Benes N.E. (2019). Swelling of 9 Polymers Commonly Employed for Solvent-Resistant Nanofiltration Membranes: A Comprehensive Dataset. J. Membrane Sci..

[27] Bobbitt N.S., Mendonca M.L., Howarth A.J., Islamoglu T., Hupp J.T., Farha O.K., Snurr R.Q. (2017). Metal-Organic Frameworks for the Removal of Toxic Industrial Chemicals and Chemical Warfare Agents. R. Soc. Chem..

[28] Hodzic A. (2004). Chapter 12: Re-Use, Recycling and Degradation of Composites. Green Composites: Polymer Composites and the Environment.

[29] Alvarado J., Acosta G., Perez F. (2016). Study of the Effect of the Dispersion of Functionalized Nanoparticles TiO_2_ with Photocatalytic Activity In LDPE. Polym. Degrad. Stab..

[30] Nikafshar S., Zabihi O., Ahmadi M., Mirmohseni A., Taseidifar M., Naebe M. (2017). The Effect of UV Light on the Chemical and Mechanical Properties of a Transparent Epoxy-Diamine System in the Presence of an Organic UV Absorber. Material.

[31] Poh L., Wu Q., Chen Y., Narimissa E. (2022). Characterization of Industrial Low-Density Polyethylene: A Thermal, Dynamic Mechanical, and Rheological Investigation. Rheol. Acta.

[32] Yuan Z., Xu X.R. (2022). Surface Characteristics and Biotoxicity of Airborne Microplastics. Compr. Anal. Chem..

[33] Nguyen T.K.N., Kim H.G., Kwac L.K., Bui Q.B., Nguyen D.M., Jeong H. (2018). Titanium Dioxide-Benzophenone Hybrid As An Effective Catalyst For Enhanced Photochemical Degradation of Low Density Polyethylene. e-Polymers.

[34] Li D., Zhou L., Wang X., He L., Yang X. (2019). Effect of Crystallinity of Polyethylene with Different Densities on Breakdown Strength and Conductance Property. Materials.

[35] Tuasikal M.A., Alothman O.Y., Luqman M., Al-Zahrani S.M., Jawaid M. (2014). Influence of Natural and Accelerated Weathering on the Mechanical Properties of Low-Density Polyethylene Films. Int. J. Polym. Anal. Charact..

[36] Sadrnia H., Zamanian M., Khojastehpour M., Hossein F., Thibault J. (2021). Effect of TiO_2_ Nanoparticles on Barrier and Mechanical Properties of PVA Films. J. Membr. Sci. Res..

[37] Kumosa M., Lu T., Solis-Ramos E., Yi Y. (2018). UV Degradation Model for Polymers and Polymer Matrix Composites. Polym. Degrad. Stabil..

[38] Zhao J., Cai G., Cui L., Larbi A.S., Tsavdaridis K.D. (2017). Deterioration of Basic Properties of the Materials in FRP-Strengthening RC Structures Under Ultraviolet Exposure. Polymers.

[39] Munir N. (2019). Determination of Low-Density Polyethylene Water Permeability, Transport Activation Energy and Mechanical Properties after Thermal Oxidation and Immersion in Water. Master’s Thesis.

[40] Mao F. (2008). Permeation of Hydrocarbons through Polyvinyl Chloride (PVC) and Polyethylene (PE) Pipes and Pipe Gaskets. Ph.D. Thesis.

[41] Siracusa V., Genovese L., Ingrao C., Munari A., Lotti N. (2018). Barrier Properties of Poly(Propylene Cyclohexanedicarboxylatee) Random Eco-Friendly Copolyesters. Polymers.

[42] Fallis I.A., Griffiths P.C., Cosgrove T., Dreiss C.A., Govan N., Heenan R.K. (2009). Locus Specific Microemulsion Catalysts for Sulfur Mustard (HD) Chemical Warfare Agent Decontamination. J. Am. Chem. Soc..

[43] Liu Y., Howarth A.J., Hupp T., Farha O.K. (2015). Selective Photooxidation of a Mustard-Gas Simulant Catalyzed by a Porphyrinic Metal-Organic Framework. Angew. Chem. Int. Ed. Engl..

[44] Hiscock J.R., Bustone G.P., Clark E.R. (2017). Decontamination and Remediation of the Sulfur Mustard Simulant CEES with “ Off-the-Shelf” Reagents in Solution and Gel States: A Proof-of-Concept Study. Chem. Open.

[45] Ergerton T.A., Yang R., Christensen P.A., White J.R., Malt A. (2011). Spectroscopic Studies of Photodegradation of Polyethylene Films Containing TiO_2_ Nanoparticles. J. App. Polym. Sci..

[46] Yang R., Christensen P.A., Egerton T.A., White J.R. (2010). Degradation Products Formed During UV Exposure of Polyethylene-ZnO Nano-Composites. Polym. Degrad. Stabil..

